# Adrenal insufficiency in a woman secondary to standard-dose inhaled fluticasone propionate therapy

**DOI:** 10.1530/EDM-13-0080

**Published:** 2014-02-01

**Authors:** Casey M Hay, Daniel I Spratt

**Affiliations:** 1 Department of Obstetrics and GynecologyMaine Medical Center22 Bramhall Street, Portland, Maine, 04102USA; 2 Division of Reproductive Endocrinology and InfertilityMaine Medical Center22 Bramhall Street, Portland, Maine, 04102USA

## Abstract

A 55-year-old woman with asthma presented with adrenal insufficiency of unknown origin. She was referred to our Division of Reproductive Endocrinology to further evaluate an undetectable morning cortisol level discovered during the evaluation of a low serum DHEA-S level. She was asymptomatic other than having mild fatigue and weight gain. Her medication list included 220 μg of inhaled fluticasone propionate twice daily for asthma, which she was taking as prescribed. On presentation, the undetectable morning cortisol level was confirmed. A urinary measurement of fluticasone propionate 17β-carboxylic acid was markedly elevated. Fluticasone therapy was discontinued and salmeterol therapy initiated with supplemental hydrocortisone. Hydrocortisone therapy was discontinued after 2 months. A repeat urinary fluticasone measurement 4 months after the discontinuation of fluticasone therapy was undetectably low and morning cortisol level was normal at 18.0 μg/dl. Inhaled fluticasone is generally considered to be minimally systemically absorbed. This patient's only clinical evidence suggesting adrenal insufficiency was fatigue accompanying a low serum DHEA-S level. This case demonstrates that adrenal insufficiency can be caused by a routine dose of inhaled fluticasone. Missing this diagnosis could potentially result in adrenal crisis upon discontinuation of fluticasone therapy.

**Learning points:**

- Standard-dose inhaled fluticasone can cause adrenal insufficiency.
- Adrenal insufficiency should be considered in patients taking, or who have recently discontinued, inhaled fluticasone therapy and present with new onset of nonspecific symptoms such as fatigue, weakness, depression, myalgia, arthralgia, unexplained weight loss, and nausea that are suggestive of adrenal insufficiency.
- Adrenal insufficiency should be considered in postoperative patients who exhibit signs of hypoadrenalism after fluticasone therapy has been withheld in the perioperative setting.
- Routine screening for hypoadrenalism in patients without clinical signs or symptoms of adrenal insufficiency after the discontinuation of inhaled fluticasone therapy is not indicated due to the apparently low incidence of adrenal insufficiency caused by fluticasone.

## Background

Adrenal suppression is a well-known consequence of chronic administration of pharmacologic doses of glucocorticoids. Routine doses of inhaled corticosteroids (ICS) are generally felt to have a minimal impact on the adrenal axis, except in certain subsets of the population such as children and patients on specific medications such as ritonavir (1, 2). It has been suggested that inhaled fluticasone propionate has an improved risk:benefit ratio compared with other ICS, probably due to a very high first-pass metabolism (3). The safety of inhaled fluticasone with respect to adrenal axis suppression has been supported by one study of 500 severe asthmatics, which showed that high-dose inhaled fluticasone (1–2 mg daily) did not decrease morning cortisol levels outside of the normal range (4). However, a large meta-analysis showed that inhaled fluticasone exhibited a greater dose-related systemic bioactivity compared with other available ICS, particularly at doses above 0.8 mg/day (5).

Herein, we report on a 55-year-old woman with profound adrenal axis suppression caused by routine doses of inhaled fluticasone. Given the prevalence of fluticasone prescriptions, it is likely that other cases of adrenal suppression with inhaled fluticasone occur. According to a 2011 review of the use of medications in the USA, published by the IMS Institute for Healthcare Informatics, medications containing fluticasone are the 16th most prescribed medications in the country. In 2011, there were 38.4 million prescriptions dispensed containing fluticasone. If even a small proportion of patients using fluticasone have resulting undiagnosed adrenal insufficiency, there could be significant health consequences.

## Case presentation

A 55-year-old woman was referred to our Reproductive Endocrinology Clinic to further evaluate an undetectable morning serum cortisol level. Initial concern for adrenal insufficiency had been raised 5 years earlier during evaluation for low bone density when a serum DHEA-S level was measured and found to be ‘low’ by the patient's history. Earlier this year, the patient's primary gynecologist observed ‘worsening’ osteopenia and referred her to an endocrinologist. Metabolic bone evaluation was normal. However, the patient mentioned her history of low DHEA-S level, prompting an evaluation of morning cortisol level, which was found to be undetectably low. She subsequently underwent two cosyntropin stimulation tests and both tests revealed baseline morning serum cortisol and adrenocorticotropic hormone (ACTH) levels below the assay detection limits (1.5 μg/dl for cortisol and 5 pg/ml for ACTH) consistent with central hypoadrenalism. Cortisol levels remained undetectably low after cosyntropin stimulation. Serum thyroxine (6.1 μg/dl), thyroid-stimulating hormone (TSH; 2.03 μU/ml), and insulin-like growth factor 1 (101 ng/ml) measurements were normal as was a chemistry panel including tests for renal and hepatic functions and fasting glucose (79 mg/dl). Follicle-stimulating hormone (FSH) levels were in the menopausal range (66.9 IU/l). Dual energy X-ray absorptiometry (DEXA) bone density measurements demonstrated *T*-scores of the spine, femoral neck, and total hip of −2.4, −1.9, and −2.1. Spinal *Z*-score was −1.3. Spinal BMD was stable between 2009 and 2012 (0.794 and 0.785 g/cm^2^). She was placed on hydrocortisone supplementation, but she did not tolerate it due to malaise. At this time, she presented to our clinic for further evaluation. She reported only mild fatigue with mild weight gain and denied having nausea, increased skin pigmentation, or postural symptoms. Symptoms of hypoadrenalism were notably absent, despite the very low cortisol levels. She also reported no symptoms of Cushing's syndrome other than mild weight gain and fatigue and had no symptoms suggestive of cortisol excess on physical examination. Past medical history was relevant for asthma controlled with 220 μg of inhaled fluticasone twice daily (a dose of 110 twice daily initiated in May 2001 and increased to 220 μg in February 2009), ipratropium bromide, and levalbuterol. She denied having any other health problems or using steroids with no history of autoimmune or pituitary disease, hepatic or renal disease, or pulmonary disease other than asthma.

## Investigation

Our initial evaluation included the confirmation of persistent suppression of ACTH and cortisol levels, evaluation of other pituitary functions with measurements of FSH and TSH levels, a complete metabolic panel, measurement of serum aldosterone levels, screening of serum and urine for exogenous glucocorticoids, and magnetic resonance imaging of the pituitary and hypothalamus. Undetectably low morning serum cortisol and ACTH levels were confirmed. All other results were within normal limits, except for the urinary measurement of fluticasone propionate 17β-carboxylic acid, which was markedly elevated at 7060 pg/ml. While there is no standard reference range for this laboratory, 10 pg/ml is considered average. Repeat testing confirmed the elevated urinary fluticasone metabolite measurement with a value of 2500 pg/ml. The patient confirmed that she was taking only the prescribed dose of inhaled fluticasone at the prescribed frequency.

## Treatment

Fluticasone therapy was discontinued, and the patient was started on salmeterol therapy for asthma control. Hydrocortisone was administered at a dose of 10 mg in the morning and 5 mg in the afternoon.

## Outcome and follow-up

Repeat morning cortisol measurements 2 weeks and 2 months after the discontinuation of fluticasone therapy were 4.0 and 4.2 μg/dl with the afternoon hydrocortisone dose being held on the day before testing. The patient discontinued hydrocortisone treatment at that time, and 3 weeks later, the morning cortisol level had risen to 8.1 μg/dl. A repeat urinary fluticasone propionate 17β-carboxylic acid measurement was undetectably low. The final morning cortisol level 4 months later was 18.0 μg/dl.

## Discussion

There have been some case reports of ICS causing adrenal suppression and crisis in adult patients receiving high-dose therapy without ritonavir (for fluticasone doses ≥1 g daily) (5) (6) (7) (8) (9) (10). Our patient is notable because she presented with complete adrenal axis suppression in the setting of standard-dose inhaled fluticasone therapy in the absence of ritonavir therapy or any other predisposing factors. She was asymptomatic other than having fatigue and underwent an extensive evaluation before establishing the correct diagnosis, indicating that this occurrence may currently be difficult to recognize and therefore underdiagnosed.

The mechanism of increased fluticasone absorption in our patient is unclear. She had no evidence of pulmonary or vascular disease. She was taking no other medications that would enhance absorption. The mechanism of adrenal axis suppression by ritonavir is the impairment of the cytochrome p450 system, leading to the accumulation of fluticasone in the blood. However, in our patient, fluticasone propionate 17β-carboxylic acid, which is the inactive metabolite of fluticasone propionate, was present at high levels consistent with intact cytochrome p450. Thus, no specific mechanism that would enable the identification of a subpopulation of patients receiving fluticasone who are more likely to have adrenal axis suppression could be identified in our patient.

This case report illustrates the potential for significant adrenal insufficiency due to standard doses of inhaled fluticasone and emphasizes the need to consider this diagnosis in patients taking ICS, particularly when glucocorticoid therapy is being discontinued. The lack of clinical symptoms of hypoadrenalism in the patient despite the consistently undetectable morning cortisol levels was most probably due to the glucocorticoid effects of the absorbed fluticasone. Recognition of the possibility of adrenal axis suppression by standard-dose inhaled fluticasone is important in two contexts. First, in patients similar to our patient whose adrenal status is being evaluated for reasons not related to their fluticasone use (e.g. fatigue, weight loss or gain, and low DHEA-S levels), the possibility of adrenal axis suppression should be considered. The reportedly low DHEA-S level in our patient while taking 110 μg of fluticasone twice daily appropriately prompted an evaluation of her adrenal axis, although the DHEA-S finding may have been incidental. Although our patient did not exhibit hyperglycemia, low *Z*-scores on DEXA, or suppression of TSH or FSH secretion, these factors should also prompt an investigation of adrenal status in patients receiving fluticasone therapy. If central adrenal insufficiency is evident, inhaled fluticasone should promptly be considered as an etiology to avoid a potentially expensive and confusing evaluation. Second and more importantly, adrenal insufficiency should be suspected in any patient who is discontinuing inhaled fluticasone therapy and developing clinical symptoms of hypoadrenalism such as unusual fatigue. Recognition of adrenal insufficiency may be difficult because symptoms are nonspecific and may include weakness, myalgia, arthralgia, nausea, weight loss, and psychiatric symptoms in addition to fatigue. Furthermore, the lack of symptoms of adrenal insufficiency in our patient despite consistently undetectable morning cortisol levels suggests that excess circulating fluticasone may induce glucocorticoid effects that prevent the symptoms of glucocorticoid deficiency. Testing for adrenal insufficiency should also be conducted when fluticasone therapy is withheld in the perioperative setting if a patient's hemodynamics or other factors suggest hypoadrenalism. Routine screening for adrenal insufficiency in patients without symptoms suggestive of hypoadrenalism after the discontinuation of inhaled fluticasone therapy is not warranted because the incidence of adrenal suppression due to inhaled fluticasone therapy appears to be low.

## Patient consent

Written informed consent was obtained from the patient for publication of this article.

## Author contribution statement

Dr D I Spratt is the patient's primary endocrinologist and contributed to the writing of this case report. Dr C M Hay is an obstetrics and gynecology resident who also directly cared for the patient and contributed to the writing of this case report.
